# The nuclear localization of SWI/SNF proteins is subjected to oxygen regulation

**DOI:** 10.1186/2045-3701-2-30

**Published:** 2012-08-29

**Authors:** Ranita Ghosh Dastidar, Jagmohan Hooda, Ajit Shah, Thai M Cao, Robert Michael Henke, Li Zhang

**Affiliations:** 1 Department of Molecular and Cell Biology, Center for Systems Biology, University of Texas at Dallas, Mail Stop RL11 800 W Campbell Road, Richardson, TX, 75080, USA

**Keywords:** Hypoxia response, Oxygen regulation, SWI/SNF, Live cell imaging, Protein localization

## Abstract

**Background:**

Hypoxia is associated with many disease conditions in humans, such as cancer, stroke and traumatic injuries. Hypoxia elicits broad molecular and cellular changes in diverse eukaryotes. Our recent studies suggest that one likely mechanism mediating such broad changes is through changes in the cellular localization of important regulatory proteins. Particularly, we have found that over 120 nuclear proteins with important functions ranging from transcriptional regulation to RNA processing exhibit altered cellular locations under hypoxia. In this report, we describe further experiments to identify and evaluate the role of nuclear protein relocalization in mediating hypoxia responses in yeast.

**Results:**

To identify regulatory proteins that play a causal role in mediating hypoxia responses, we characterized the time courses of relocalization of hypoxia-altered nuclear proteins in response to hypoxia and reoxygenation. We found that 17 nuclear proteins relocalized in a significantly shorter time period in response to both hypoxia and reoxygenation. Particularly, several components of the SWI/SNF complex were fast responders, and analysis of gene expression data show that many targets of the SWI/SNF proteins are oxygen regulated. Furthermore, confocal fluorescent live cell imaging showed that over 95% of hypoxia-altered SWI/SNF proteins accumulated in the cytosol in hypoxic cells, while over 95% of the proteins were nuclear in normoxic cells, as expected.

**Conclusions:**

SWI/SNF proteins relocalize in response to hypoxia and reoxygenation in a quick manner, and their relocalization likely accounts for, in part or in whole, oxygen regulation of many SWI/SNF target genes.

## Background

Living organisms ranging from yeast to mammals use oxygen to generate their cellular energy supply and to synthesize important biomolecules. Hence, they need to respond effectively to changes in oxygen levels in the environment, particularly to hypoxia [1,2]. In humans, hypoxia is responsible for death or damage by the ischemia accompanying heart attack, stroke, and traumatic injuries [3-5]. The molecular and cellular events induced by changes in oxygen levels are very broad in eukaryotes. For example, over 20% of yeast genes change their transcript levels in response to hypoxia [6]. In the human arterial endothelial cells, more than 8% of all genes alter their transcript levels by at least 1.5-fold in response to hypoxia [7]. In the human primary astrocytes, more than 5% of the genes alter their transcript levels by at least 2-fold in response to hypoxia [8]. Such broad changes in gene expression likely involve coordinated actions of multiple pathways and regulators.

Previous studies have identified several transcriptional regulators, including Mga1 and Rox1, that can mediate oxygen regulation of gene expression in the yeast *Saccharomyces cerevisiae*[9,10]. However, these regulators can account for the regulation of only a fraction of hypoxia-regulated genes [6]. Many other regulators are likely involved in mediating oxygen regulation. Recently, in an effort to systematically identify proteins that can mediate oxygen regulation and signaling, we performed a genome-wide screen for proteins that exhibit altered cellular distribution patterns in response to hypoxia and reoxygenation [11]. We found that over 200 proteins alter their cellular locations in response to hypoxia. Particularly, under hypoxia, a good number (at least 121) of nuclear proteins do not localize to the nucleus, but accumulate in the cytosol. In response to reoxygenation, they readily localize to the nucleus. Notably, many of these hypoxia-redistributed nuclear proteins are subunits of key regulatory complexes involved in chromatin remodeling (such as the SWI/SNF complex) [12-14], in transcriptional regulation (such as the SAGA complex) [15], and in splicing (such as the MRP complex) [16]. Hence, it is conceivable that some of these complexes can play a dominant role in mediating oxygen regulation of gene expression.

To further assess the roles of these regulators in mediating oxygen signaling and regulation, we examined the time course characteristics of the relocalization of these proteins in response to hypoxia and reoxygenation. We found a small group of nuclear proteins relocalized in a significantly shorter time period in response to both hypoxia and reoxygenation, when compared to other proteins. These proteins include three components of the SWI/SNF complex. Furthermore, using confocal fluorescent imaging of live cells, we quantitatively characterized the effect of hypoxia on the distribution of SWI/SNF proteins. We found that in live hypoxic cells, over 95% of Swi3, Snf5, Snf6, Snf11, Snf12 and Swp82 were in the cytosol, while over 95% of hypoxia-unaffected proteins, such as Swi2 and Taf14, were in the nucleus. These results suggest that hypoxia can significantly alter the composition and property of the SWI/SNF complex and mediate oxygen regulation of gene expression.

## Results

Among the hypoxia-redistributed nuclear proteins we previously identified, some are likely involved in mediating oxygen signaling and regulation of gene expression. Particularly, proteins that change their locations in relatively shorter time periods are likely the regulators that initiate further downstream events in responses to hypoxia and reoxygenation. In other words, they are likely to be positioned in the upstream of the hierarchy of the molecular events elicited by hypoxia or reoxygenation, and are responsible for initiating downstream changes such as those in gene expression. We therefore decided to characterize the time course response of the hypoxia-redistributed nuclear proteins in response to hypoxia and reoxygenation.

**Figure 1:**
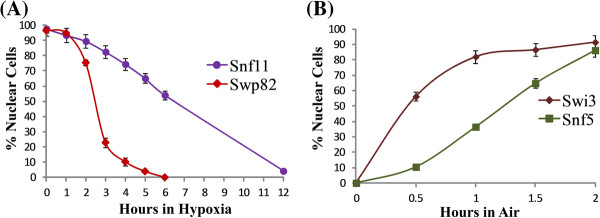
**Time course characteristics of protein relocalization elicited by hypoxia or reoxygenation.** (**A**) The time courses of relocalization of Snf11 and Swp82 in response to hypoxia. Cells expressing Snf11-GFP or Swp82-GFP were grown in air and then shifted to hypoxic growth conditions. At various time points, cells were imaged, and the number of cells showing GFP-tagged proteins in the nucleus (N) or cytosol (C) was counted. The percentage of cells showing nuclear locations is calculated and plotted. (**B**) The time courses of relocalization of Snf5 and Swi3 in response to reoxygenation. Cells expressing Snf5-GFP or Swi3-GFP were grown under hypoxia and then shifted to normoxic growth conditions. At various time points, cells were imaged, and the number of cells showing GFP-tagged proteins in the nucleus (N) or cytosol (C) was counted. The percentage of cells showing nuclear localization is plotted here.

**Figure 2:**
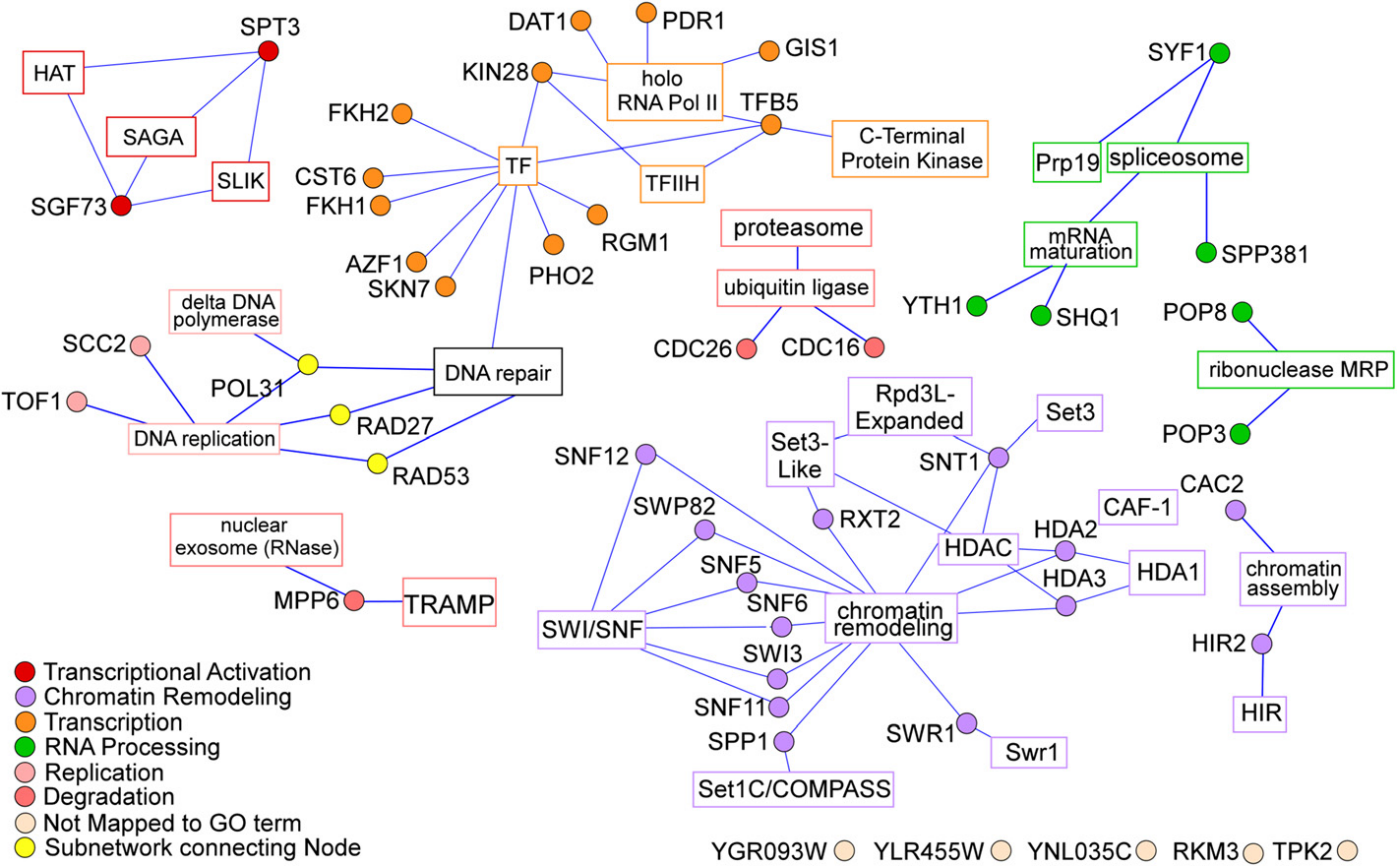
**Graphical representation of protein-protein interaction networks for the nuclear proteins that localized to the cytosol in response to hypoxia in a shorter time period.** The information on the biochemical interactions and complex formation of the 48 faster responding nuclear proteins (listed in Table 1) was downloaded from the SGD database, and then imported to Cytoscape for network construction. The proteins are shown as round nodes in different colors based on their cellular functions. The GO terms for protein complexes or functional categorizations are indicated and are shown in square nodes. Nodes of the same sub-networks are colored similarly, and a key for the coloring of the nodes is shown. Lines represent an association of the protein to a particular complex or functional GO term.

First, we examined the time course characteristics of nuclear proteins in response to hypoxia. We found that all proteins became predominantly cytosolic after exposure to hypoxia for 12 hours; see Snf11 in Figure 1A for an example. One group of these proteins became predominantly cytosolic after only 6 hours or shorter times; see Swp82 in Figure 1A for an example. This group has 48 proteins (see Table 1). They include several transcriptional regulators and regulators of chromatin, DNA replication and repair, and RNA processing (Figure 2). Notably, five components of the SWI/SNF complex relocalized in 6 hours (see Table 1 and Figure 2), suggesting that they may have a signaling role in initiating downstream events.

**Table 1:** Nuclear proteins that relocalized to the cytosol in response to hypoxia in a shorter time period

**ORF name**	**Gene name**	**Description**
YOR113W	AZF1	Involved in glucose induction of CLN3 transcription
YML102W	CAC2	Component of the chromatin assembly complex
YKL022C	CDC16	Subunit of the anaphase-promoting complex/cyclosome
YFR036W	CDC26	Subunit of the Anaphase-Promoting Complex/Cyclosome
YIL036W	CST6	Member of the ATF/CREB family
YML113W	DAT1	DNA binding protein that recognizes oligo(dA).oligo(dT) tracts
YIL131C	FKH1	Forkhead family transcription factor
YNL068C	FKH2	Forkhead family transcription factor
YDR096W	GIS1	JmjC domain-containing histone demethylase
YDR295C	HDA2	Subunit of a class II histone deacetylase complex
YPR179C	HDA3	Subunit of a class II histone deacetylase complex
YOR038C	HIR2	Subunit of the HIR nucleosome assembly complex
YDL108W	KIN28	Subunit of the transcription factor TFIIH
YNR024W	MPP6	RNA binding protein that associates with the exosome
YGL013C	PDR1	Master regulator of multidrug resistance genes
YDL106C	PHO2	Homeobox transcription factor
YJR006W	POL31	DNA polymerase III (delta) subunit
YNL282W	POP3	Subunit of both RNase MRP
YBL018C	POP8	Subunit of both RNase MRP
YKL113C	RAD27	5' to 3' exonuclease, 5' flap endonuclease
YPL153C	RAD53	Required for cell-cycle arrest in response to DNA damage
YMR182C	RGM1	Putative transcriptional repressor
YBR095C	RXT2	Subunit of the histone deacetylase Rpd3L complex
YDR180W	SCC2	Subunit of cohesin loading factor (Scc2p-Scc4p)
YGL066W	SGF73	Subunit of SAGA histone acetyltransferase complex
YIL104C	SHQ1	Required for the assembly of box H/ACA snoRNPs
YHR206W	SKN7	Regulator for optimal induction of heat-shock genes
YDR073W	SNF11	Subunit of the SWI/SNF chromatin remodeling complex
YNR023W	SNF12	73 kDa subunit of the SWI/SNF chromatin remodeling complex
YBR289W	SNF5	Subunit of the SWI/SNF chromatin remodeling complex
YHL025W	SNF6	Subunit of the SWI/SNF chromatin remodeling complex
YCR033W	SNT1	Subunit of the Set3C deacetylase complex
YPL138C	SPP1	Subunit of the COMPASS complex
YBR152W	SPP381	Component of U4/U6.U5 tri-snRNP
YDR464W	SPP41	Negative regulator of expression of PRP4 and PRP3
YDR392W	SPT3	Subunit of the SAGA and SAGA complexes
YJL176C	SWI3	Subunit of the SWI/SNF chromatin remodeling complex
YFL049W	SWP82	Subunit of the SWI/SNF chromatin remodeling complex
YDR334W	SWR1	Component of the SWR1 complex
YDR416W	SYF1	Component of the spliceosome complex
YDR079C-A	TFB5	Component of TFIIH
YNL273W	TOF1	Subunit of a replication-pausing checkpoint complex
YPL203W	TPK2	cAMP-dependent protein kinase catalytic subunit
YBR030W	YBR030W	Putative ribosomal lysine methyltransferase
YGR093W	YGR093W	Putative debranching enzyme associated ribonuclease
YLR455W	YLR455W	Putative protein of unknown function
YNL035C	YNL035C	Putative protein of unknown function
YPR107C	YTH1	Component of cleavage and polyadenylation factor

**Figure 3:**
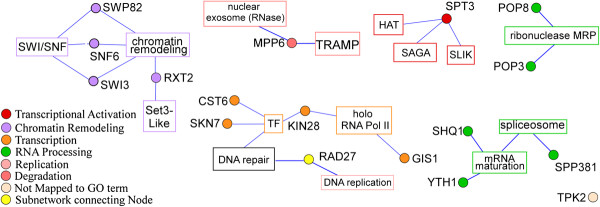
**Graphical representation of protein-protein interaction networks for the nuclear proteins that changed their locations in response to hypoxia and reoxygenation in shorter time periods.** The information on the biochemical interactions and complex formation of the 17 faster responding nuclear proteins was downloaded from the SGD database, and then imported to Cytoscape for network construction. The proteins are shown as round nodes in different colors based on their cellular functions. The GO terms for protein complexes or functional categorizations are indicated and shown in square nodes. Nodes of the same sub-networks are colored similarly, and a key for the coloring of the nodes is shown. Lines represent an association of the protein to a particular complex or functional GO term.

Second, we characterized the changes in protein distribution when cells grown under hypoxia were exposed to oxygen. We found that 76 hypoxia-redistributed nuclear proteins (see Table 2) recovered their nuclear locations in the majority of cells in one hour; see Swi3 in Figure 1B for an example. The rest of the proteins recovered their nuclear location in the majority of the cells in 2 or more hours; see Snf5 in Figure 1B for an example. Among these nuclear proteins, 17 of them responded to both hypoxia and reoxygenation in shorter times than the rest of the proteins (see Figure 3). Notably, 3 of these faster responding proteins are components of the SWI/SNF complex (Figure 3). These and previous results strongly suggest that the SWI/SNF proteins play regulatory roles in mediating oxygen regulation and hypoxia response. Given their roles in chromatin remodeling and transcriptional regulation [17,18], they are likely responsible for initiating certain changes in gene expression in response to changes in oxygen levels. Although 14 other proteins also responded to hypoxia and reoxygenation in shorter times, they are generally not components of one regulatory complex (Figure 3).

**Table 2:** Proteins that recovered their nuclear locations in response to oxygen in a shorter time period

**ORF name**	**Gene name**	**Description**
YBR236C	ABD1	Methyltransferase
YPR180W	AOS1	Smt3p (SUMO) activator
YJL115W	ASF1	Nucleosome assembly factor
YNR010W	CSE2	Subunit of the RNA polymerase II mediator complex
YIL036W	CST6	Member of the ATF/CREB family
YJL006C	CTK2	Beta subunit of C-terminal domain kinase I
YEL018W	EAF5	Subunit of the NuA4 acetyltransferase complex
YMR277W	FCP1	Carboxy-terminal domain (CTD) phosphatase
YCL011C	GBP2	Poly(A+) RNA-binding protein
YGR252W	GCN5	Subunit of the ADA and SAGA complexes
YDR096W	GIS1	JmjC domain-containing histone demethylase
YDR174W	HMO1	Chromatin associated high mobility group family member
YFL013C	IES1	Subunit of the INO80 chromatin remodeling complex
YHR085W	IPI1	Essential component of the Rix1 complex
YIL026C	IRR1	Subunit of the cohesin complex
YDL108W	KIN28	Subunit of the transcription factor TFIIH
YDL087C	LUC7	Associated with the U1 snRNP complex
YMR043W	MCM1	Involved in cell-type-specific transcription
YDL005C	MED2	Subunit of the RNA polymerase II mediator complex
YMR070W	MOT3	Nuclear transcription factor mediating hypoxia response
YKL059C	MPE1	Essential conserved subunit of CPF
YNR024W	MPP6	Nuclear RNA binding protein
YLR116W	MSL5	Component of the commitment complex
YPR144C	NOC4	Mediating maturation and nuclear export of 40S
YHR133C	NSG1	Regulator of sterol biosynthesis
YKR082W	NUP133	Subunit of the nuclear pore complex
YAR002W	NUP60	Subunit of the nuclear pore complex
YJL061W	NUP82	Nucleoporin, subunit of the nuclear pore complex (NPC)
YOL115W	PAP2	Catalytic subunit of TRAMP
YDR228C	PCF11	mRNA 3' end processing factor
YMR076C	PDS5	Required for sister chromatid condensation and cohesion
YNL282W	POP3	Subunit of both RNase MRP
YGR030C	POP6	Subunit of both RNase MRP
YBL018C	POP8	Subunit of both RNase MRP
YLL036C	PRP19	Splicing factor associated with the spliceosome
YGR156W	PTI1	Pta1p Interacting protein
YKL113C	RAD27	5' to 3' exonuclease, 5' flap endonuclease
YGL246C	RAI1	Required for pre-rRNA processing
YNL216W	RAP1	Involved in either activation or repression of transcription
YDR195W	REF2	RNA-binding protein
YAR007C	RFA1	Subunit of heterotrimeric Replication Protein A
YNL290W	RFC3	Subunit of heteropentameric Replication factor C
YOL094C	RFC4	Subunit of heteropentameric Replication factor C
YHR197W	RIX1	Essential component of the Rix1 complex
YMR061W	RNA14	Cleavage and polyadenylation factor I (CF I) component
YJL011C	RPC17	RNA polymerase III subunit C17
YER117W	RPL23B	Component of the large (60S) ribosomal subunit
YDR427W	RPN9	Non-ATPase regulatory subunit of the 26S proteasome
YHR062C	RPP1	Subunit of both RNase MRP
YBR095C	RXT2	Subunit of the histone deacetylase Rpd3L complex
YIL084C	SDS3	Component of the Rpd3p/Sin3p deacetylase complex
YJL168C	SET2	Histone methyltransferase
YIL104C	SHQ1	Required for the assembly of box H/ACA snoRNPs
YHR206W	SKN7	Regulator of heat-shock genes
YGR074W	SMD1	Core Sm protein Sm D1
YHL025W	SNF6	Subunit of the SWI/SNF chromatin remodeling complex
YMR016C	SOK2	Involved in the cAMP-dependent protein kinase signaling
YBR152W	SPP381	mRNA splicing factor
YER161C	SPT2	Involved in negative regulation of transcription
YDR392W	SPT3	Subunit of the SAGA and SAGA-like complexes
YIL143C	SSL2	Component of RNA polymerase transcription factor TFIIH
YBR231C	SWC5	Component of the SWR1 complex
YJL176C	SWI3	Subunit of the SWI/SNF chromatin remodeling complex
YFL049W	SWP82	Subunit of the SWI/SNF chromatin remodeling complex
YGR129W	SYF2	Component of the spliceosome complex
YGR274C	TAF1	TFIID subunit (145 kDa)
YGL112C	TAF6	Subunit (60 kDa) of TFIID and SAGA complexes
YDR311W	TFB1	Subunit of TFIIH and nucleotide excision repair factor complexes
YPL203W	TPK2	cAMP-dependent protein kinase catalytic subunit
YDR165W	TRM82	Subunit of a tRNA methyltransferase complex
YNL246W	VPS75	NAP family histone chaperone
YOR229W	WTM2	Regulator of meiosis, silencing, and expression of RNR genes
YHR090C	YNG2	Subunit of the NuA4 histone acetyltransferase complex
YIL063C	YRB2	Involved in nuclear processes of the Ran-GTPase cycle
YGR270W	YTA7	Regulator of histone gene expression
YPR107C	YTH1	Component of cleavage and polyadenylation factor

**Figure 4:**
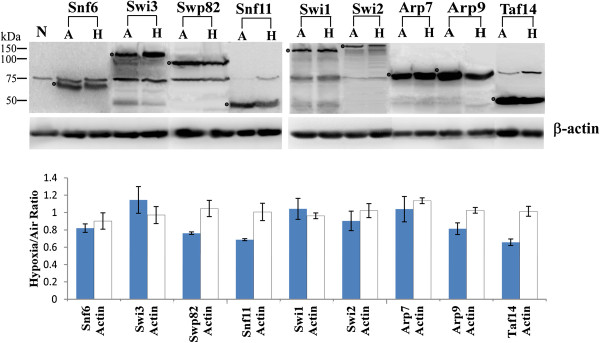
**Western blot showing TAP-tagged proteins in extracts prepared from normoxic and hypoxic cells.** Shown here are proteins in extracts from the parent BY4741 cells without any TAP-tagged proteins expressed (N), and from cells grown in air (A) or under hypoxia (H) which expresses Snf6-TAP (molecular mass: 58 kDa), Swi3-TAP (113 kDa), Swp82-TAP (90 kDa), Snf11-TAP (40 kDa), Swi1-TAP (168 kDa), Swi2-TAP (214 kDa), Arp7-TAP (74 kDa), Arp9-TAP (73 kDa), Taf14-TAP (47 kDa), respectively. For the hypoxic condition, cells were placed in a hypoxia chamber for up to 12 hours (the time period necessary for the proteins to relocate to the cytosol). The intensity of bands representing the Swi/Snf proteins was quantified, and the intensity ratios of the bands representing the Swi/Snf proteins in hypoxic vs. normoxic cells were plotted and shown below the Western blot images. The data plotted are averages of three replicates.

Therefore, we decided to further characterize the effect of hypoxia on the SWI/SNF proteins. First, we examined if changes in oxygen levels affect the protein levels of SWI/SNF proteins. To this end, we detected and compared the levels of SWI/SNF proteins in hypoxic and normoxic cells. We used yeast strains expressing the SWI/SNF proteins with the TAP tag at the C-terminus from the natural chromosomal locations [19]. We found that the levels of all detected SWI/SNF proteins were not significantly affected by hypoxia (Figure 4). The variations in the ratios of protein levels in hypoxic vs. normoxic cells were generally less than 30%, suggesting that hypoxia did not cause significant degradation of the Swi/Snf proteins during the time period when the proteins would be relocalized to the cytosol. These proteins include those whose cellular location was affected by hypoxia, such as Snf6, Swi3, Swp82 and Snf11 (see Figure 4). They also include all those SWI/SNF proteins whose localization was not affected by hypoxia. These results show that the levels of SWI/SNF proteins are not regulated by oxygen levels.

**Figure 5:**
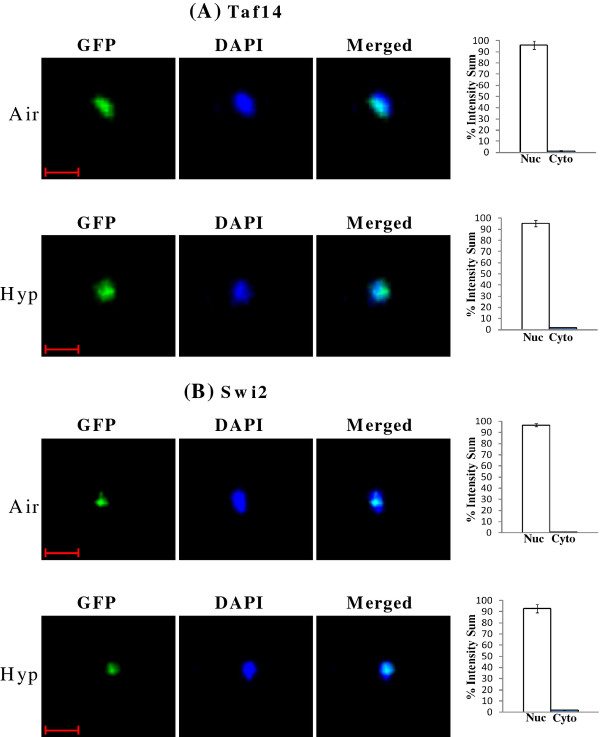
**Examples of GFP, DAPI and merged confocal fluorescent images of cells expressing proteins whose cellular localization is not affected by hypoxia.** Cells expressing Taf14-GFP (**A**) or Swi2-GFP (**B**) were grown in air or under hypoxia (Hyp), and the images were captured. The percentages of GFP fluorescence in the nucleus (N) or cytosol (C) were quantified and plotted here. The scale bar represents 1 μm.

**Figure 6:**
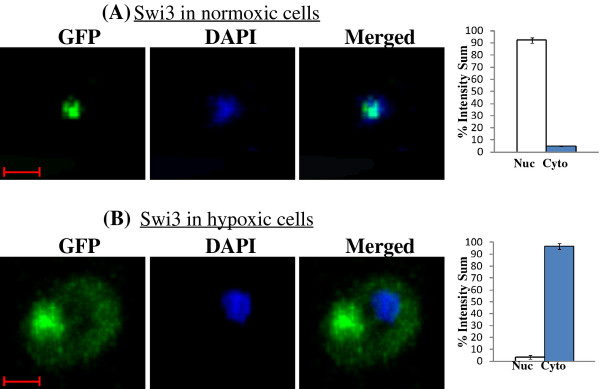
**DAPI and merged confocal fluorescent images of cells expressing Swi3-GFP.** Cells were grown in air or under hypoxia (Hyp), and the images were captured. The percentages of GFP fluorescence in the nucleus (N) or cytosol (C) was quantified and plotted here. The scale bar represents 1 μm.

**Figure 7:**
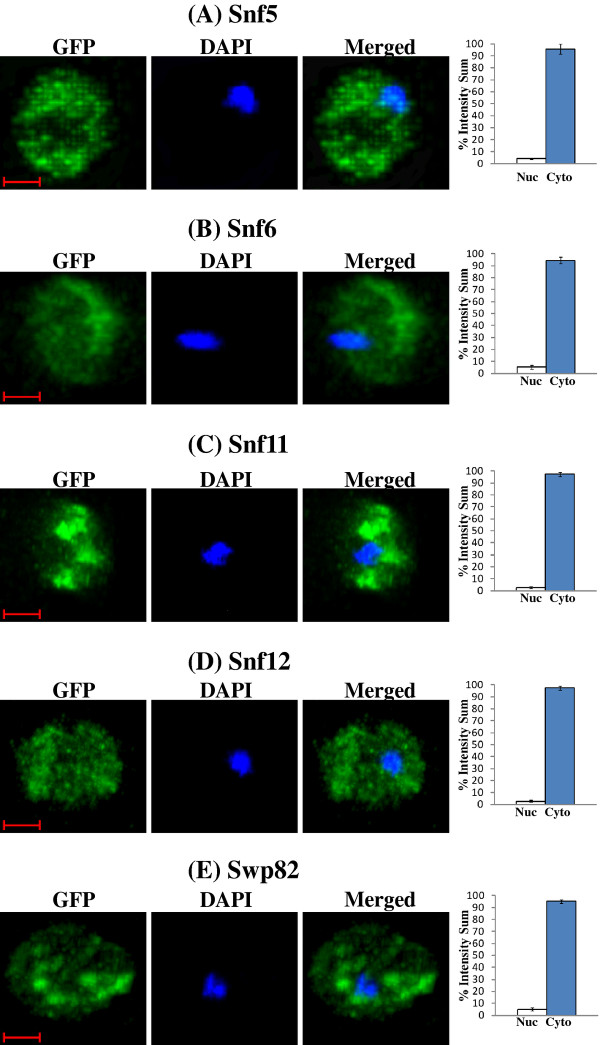
**DAPI and merged confocal fluorescent images of cells expressing SWI/SNF proteins whose cellular location is affected by hypoxia.** Cells expressing Snf5-GFP, Snf6-GFP, Snf11-GFP, Snf12-GFP and Swp82-GFP were grown in air or under hypoxia (Hyp), and the images were captured. Only the images of hypoxic cells are shown, because the normoxic cells all exhibit the same nuclear pattern as shown in Figures 5 and 6. The percentages of GFP fluorescence in the nucleus (N) or cytosol (C) were quantified and plotted here. The scale bar represents 1 μm.

Therefore, the regulation of nuclear localization is likely the dominant mechanism mediating oxygen regulation of SWI/SNF proteins and the regulation of their targets. To further confirm the regulation of nuclear localization of the SWI/SNF proteins by oxygen, we quantitatively examined and compared their distribution in live hypoxic and normoxic cells, by using confocal fluorescent live cell imaging. As expected, for the SWI/SNF proteins whose localization was not affected by oxygen levels, over 95% of the proteins was present in the nucleus in both normoxic and hypoxic cells (see Figure 5). Figure 5A shows the distribution of Taf14 in air and under hypoxia, while Figure 5B shows the distribution of Swi2. For those proteins whose localization was affected by oxygen, over 95% of the proteins was present in the nucleus in air, whereas over 95% of the proteins was present in the cytosol under hypoxia (Figures 6 and 7). Figure 6A shows the distribution of Swi3 in normoxic cells, while Figure 6B shows the distribution of Swi3 in hypoxic cells. We also quantified the distribution of other hypoxia-relocalized SWI/SNF proteins (Figure 7). Figure 7A-E show the distribution of Snf5, Snf6, Snf11, Snf12 and Swp82 in hypoxic cells (The images for normoxic cells invariably showed nuclear localization, as expected and as shown in Figures 5 and 6, and are therefore omitted). Clearly, Swi2, Snf5, Snf6, Snf11, Snf12 and Swp82 proteins were transported to the nucleus in normoxic cells, but they accumulated in the cytosol in hypoxic cells.

To further ascertain the role of SWI/SNF proteins in oxygen regulation of gene expression, we determined if and how many oxygen-regulated genes are SWI/SNF protein targets as well. To this end, we used our previous microarray and computational work analyzing genes regulated by oxygen/ and Δ *hap1* cells [6]. We also used the previously identified targets of 263 transcription factors [20]. Using these two sets of data and the R program, we identified those hypoxia-regulated genes that are targets of SWI/SNF proteins and calculated the p-values. Table 3 shows that in the wild type *HAP1* cells, 95, 112, 67, 109, 9 and 19 target genes of Swi2, Swi3, Snf5, Snf6, Snf11 and Taf14, respectively, are oxygen regulated. In Δ *hap1* cells, similar numbers of these SWI/SNF targets are hypoxia altered. These results strongly suggest that SWI/SNF proteins play a major role in mediating oxygen regulation and hypoxia responses. Furthermore, the changes in the relocalization of SWI/SNF proteins in response to hypoxia are completed between 6–12 hours or 1–2 generations; and the changes in the relocalization of SWI/SNF proteins in response to reoxygenation are completed in less than one generation. In contrast, the transcriptome response to hypoxia are completed after 5–6 generations; and the transcriptome response to reoxygenation are completed in 2 generations [21]. These results show that changes in SWI/SNF protein localization precede transcriptome responses. They therefore strongly suggest that oxygen regulation of SWI/SNF protein localization contribute to, at least in part, oxygen regulation of gene expression.

**Table 3:** The number of Swi/Snf targets whose transcript level is regulated by oxygen

	***HAP1 *cells**		Δ ***hap1 *cells**	
	**Targets**	**p-value**	**Targets**	**p-value**
**Swi2**	95	7.55E-69	119	2.87E-69
**Swi3**	112	6.14E-94	118	7.31E-102
**Snf5**	67	5.24E-49	71	1.05E-53
**Snf6**	109	8.06E-73	139	1.10E-107
**Snf11**	9	6.79E-10	6	7.45E-06
**Taf14**	19	8.09E-19	20	3.0E-20

## Discussion

The SWI/SNF complex is an ATP-dependent chromatin remodeling complex [22]. Its composition and function are conserved from yeast to humans [13]. In yeast, more than 10% of the genes are the targets of SWI/SNF proteins, although the targets of different SWI/SNF proteins are different [20]. Hypoxia and reoxygenation induce changes in gene expression in over 20% of yeast genes [6]. Such broad changes in gene expression involve the action of an array of regulators. Previous studies have shown that Mga2, Rox1, Hap1 and Mot3 are all involved in mediating oxygen regulation of several subsets of genes [6,21,23,24]. In this report, we show that several SWI/SNF proteins alter their subcellular localization readily in response to hypoxia or reoxygenation and that this change in subcellular localization likely contributes to oxygen regulation of SWI/SNF target genes.

Because the targets of SWI/SNF proteins overlap but are not identical [20], it is likely that different SWI/SNF proteins act on different groups of genes and control their expression. Here, we show that six SWI/SNF proteins accumulate in the cytosol in hypoxic cells, and relocalize to the nucleus in response to reoxygenation. Furthermore, several of the SWI/SNF proteins respond to hypoxia or reoxygenation and relocalize in a relatively quick manner. The redistribution of the six SWI/SNF proteins in the cytosol should presumably change the composition, and thereby the function or selectivity of the SWI/SNF complexes in the nucleus. Hence, the relocalization can affect the target expression of not only these SWI/SNF proteins whose localization is altered by hypoxia, such as Swi3, but also those whose localization is not affected by hypoxia, such as Swi2 and Taf14 (Table 3). Very likely, under hypoxia, because Swi3 and other proteins are predominantly present in the cytosol, the nuclear SWI/SNF proteins, such as Swi2, are likely complexed with other proteins, and act as chromatin remodelers on different sets of target genes. This explains why many SWI/SNF target genes are altered by hypoxia/reoxygenation (Table 3). Previous studies showed that Swi2, Arp7 and Arp9 form a core subcomplex possessing the ATP-dependent remodeling activity [25], while Swi3 controls SWI/SNF assembly, ATP-dependent H2A-H2B displacement, as well as recruitment to target genes [25,26]. Notably, the nuclear localization of Swi2, Arp7 and Arp9 is not affected by hypoxia, while Swi3 is affected. This supports the idea that the core complex can associate with other as yet unidentified proteins and form a different kind of SWI/SNF complexes in the nucleus in hypoxic cells.

**Figure 8:**
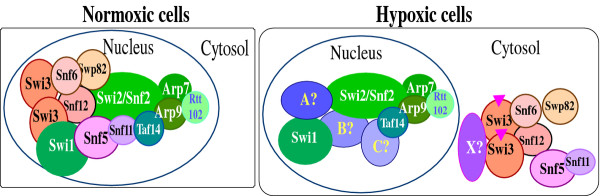
**A cartoon illustrating how oxygen may affect SWI/SNF composition and function.** In normoxic cells, the components form the SWI/SNF complex in the nucleus, enabling it to remodel chromatin at the target genes. In hypoxic cells, Swi3 and five other components are retained in the cytosol, perhaps due to modifications of these components and/or interactions with unidentified factor(s) X. In the nucleus, Swi2 and other remaining components may interact with some other proteins (marked as A, B, and C), forming complexes with different composition and targeting different sets of genes.

These results suggest a model for how oxygen may modulate SWI/SNF complex composition and function (Figure 8). In normoxic cells, SWI/SNF components form complexes in the nucleus and remodel chromatin structure at their target genes. In hypoxic cells Swi3 and other five SWI/SNF proteins accumulate in the cytosol, leaving the Swi2-Arp7-Arp9 core subcomplex available to interact with other proteins. Consequently, a different kind of SWI/SNF complex containing the core complex and other proteins (A, B and C in Figure 8) can be formed and act to remodel chromatin and control gene expression in different sets of genes. In response to reoxygenation, Swi3 and other proteins can readily relocalize to the nucleus, forming the normoxic SWI/SNF complexes, and re-establish gene expression patterns under normoxic conditions.

This model is also consistent with a recent study showing that Swi3 is a key regulator in controlling respiration genes [27]. The authors used a computational approach to analyze modules of genes with a common regulation that are affected by specific DNA polymorphisms. They integrated genotypic and expression data for individuals in a segregating population with complementary expression data of strains mutated in a variety of regulatory proteins, in order to identify regulatory-linkage modules. In so doing, they found that Swi3 is a dominant regulator in the control of respiratory gene expression [27]. The effect of *swi3* deletion is stronger than that of known respiratory regulators, including Hap2/3/4/5, Mot3 and Rox1. This is in complete agreement with our results showing that hundreds of SWI/SNF targets are altered by hypoxia (Table 3), and supports our model (Figure 8). The regulation of SWI/SNF protein localization may also occur in other eukaryotes. For example, in mammalian cells, recent studies showed that SWI/SNF proteins are important for oxygen regulation in mammalian cells [28,29]. It is likely that SWI/SNF proteins can respond to changes in oxygen levels and regulate gene expression in diverse eukaryotes.

## Conclusions

Several SWI/SNF proteins, including Swi3, Snf6 and Swp82, respond to hypoxia or reoxygenation and alter their subcellular distribution in a relatively quick manner. This change in localization likely contributes to oxygen regulation of SWI/SNF target genes.

## Methods

### Yeast strains and antibodies

The yeast GFP clone collection of 4159 strains expressing GFP-tagged proteins [30] was purchased from Invitrogen Corp. The anti-TAP monoclonal antibody was purchased from Open Biosystems.

### The creation of hypoxic growth conditions

Hypoxic (~10 ppb O_2_) growth condition was created by using a hypoxia chamber (Coy Laboratory, Inc.) and by filling the chamber with a mixture of 5% H_2_ and 95% N_2_ in the presence of a palladium catalyst [31]. The oxygen level in the chamber was monitored by using the Model 10 gas analyzer (Coy Laboratory, Inc.). The precise level of oxygen was also estimated by using a CHEMetrics rhodazine oxygen detection kit (K-7511) with the minimum detection limit at 1 ppb, and a range of 0–20 ppb. The hypoxic state was further confirmed by measuring oxygen-controlled promoter activities, including UAS1/*CYC1, ANB1* and *OLE1*[9,10,31].

### Time course characterization of cellular localization of SWI/SNF proteins

For a time course characterization of SWI/SNF protein relocalization in response to hypoxia or reoxygenation, we used a previously defined nuclear protein import assay in yeast [32-34]. Briefly, cells expressing GFP-tagged proteins at various time points of hypoxia or reoxygenation treatment were collected, and images were acquired. At least 25 cells were counted at each time point, and three sets of cells were counted. A particular cell was counted as having the GFP-tagged protein in the nucleus if the nucleus was much brighter than the surrounding cytoplasm and a clear nuclear-cytoplasmic boundary was visible. Cells with excessive bright or weak fluorescence or with aberrant morphology were not scored.

### Confocal fluorescent live cell imaging and quantitation

GFP-tagged strains were grown in synthetic complete media in air or in a hypoxia chamber. Cells were collected and subjected to confocal fluorescent imaging and quantitation. Image acquisition of live cells was performed by using a Perkin Elmer UltraView ERS Spinning Disc Confocal Microscope (Perkin Elmer, Waltham, MA) with a Zeiss 100x/1.4 Oil Immersion objective (Carl Zeiss, Thornwood, NY). High speed images were captured by using a Hamamatsu EMCCD C9100 digital camera (Hamamatsu Corporation, Bridgewater, NJ). Z-stacks were recorded for the DAPI channel (EX 405nm) and the GFP channel (EX 488nm) by moving the objective turret with a UltraView z-focus drive (Perkin Elmer, Waltham, MA). Volocity 5.4.2 (Improvision, Perkin Elmer, MA, USA) was used for image acquisition. The 3D confocal images were analyzed, and statistical data were collected by using Imaris 7.4.0 (Bitplane, South Windsor, CT).

### Preparation of yeast cell extracts and Western blotting

Yeast cells expressing various TAP-tagged proteins were grown to an optical density (OD_600_) of approximately 0.8. Cells were harvested and resuspended in 3 packed cell volumes of buffer (20 mM Tris, 10 mM MgCl_2_, 1 mM EDTA, 10% glycerol, 1 mM dithiothreitol, 0.3 M NaCl, 1 mM phenylmethylsulfonyl fluoride, 1 mg of pepstatin per ml, 1 mg of leupeptin per ml). Cells were then permeabilized by agitation with 4 packed cell volumes of glass beads, and extracts were collected as described previously [35]. Protein concentrations were determined by the BCA (bicinchoninic acid) protein assay kit (Pierce).

For Western blotting, approximately 100 μg of whole-cell extracts were first separated on 8% sodium dodecyl sulfate (SDS)–polyacrylamide gels and then transferred to polyvinylidene difluoride or nitrocellulose membranes (Bio-Rad Laboratories). TAP-tagged proteins were detected by using a monoclonal antibody against TAP and a chemiluminescence Western blotting kit (Roche Diagnostics). The signals were detected and quantified by using a Kodak image station 4000MM Pro with the molecular imaging software, version 4.5.

### Protein GO analysis and construction of the network map

The analysis of functional categories of relocalized proteins was performed on Funspec ( http://funspec.med.utoronto.ca/). For constructing the protein network map, the Cytoscape application program ( http://www.cytoscape.org/) was used. The faster responding proteins identified were mapped according to their GO terms. They were obtained by using the SGD Gene Ontology Slim Mapper Web Tool set to "Macromolecular Complex terms: Components" on the SGD website. The mapped output file was reformatted into a Cytoscape compatible network file, and the network map was created. The network map was further graphically refined by using the Canvas application program. The subcellular compartments of these proteins in normoxic cells were designated based on data from the O'Shea lab [30].

## Competing interests

The authors declare that they have no competing interests.

## Authors’ contributions

RGD, TMC and RMK performed time course studies and constructed network maps. JH and AS performed confocal fluorescent imaging and quantitation and analysis of regulator targets. LZ conceived of the study and drafted the manuscript. All authors read and approved the final manuscript.
